# Thirty years of the Association of Coloproctology of Great Britain and Ireland

**DOI:** 10.1111/codi.15356

**Published:** 2020-11-21

**Authors:** Steven R. Brown, Andrew J. Shorthouse, Paul J. Finan, the Executive of ACPGBI, Graham Branaghan, Michael Davies, Laura Hancock, Arthur Harikrishnan, Seamus Kelly, Charles Maxwell‐Armstrong, Peter Sagar, Shahab Siddiqui, James Tiernan, James Wheeler

**Affiliations:** ^1^ Sheffield Teaching Hospitals NHS FT Northern General Hospital Sheffield UK; ^2^ Sheffield Hallam University Sheffield UK; ^3^ University of Leeds Leeds UK; ^4^ Leeds Teaching Hospitals NHS Trust Leeds UK; ^5^ Association of Coloproctology of Great Britain and Ireland Lincolns Inn Fields London UK

**Keywords:** ACPGBI, history of coloproctology

## Abstract

This is a summary of the history of the Association of Coloproctology of Great Britain and Ireland from its inception in the late 1980s to the present day.

**What does this article add to the literature?**

This article summarises the history of the Association of Coloproctology of Great Britain and Ireland from its inception 30 years ago to the present day. It gives an insight into the politics of coloproctology in the UK and Ireland and how the Association has moved with the times in supporting its members.

## Introduction

The idea of an Association is to provide a focus and a forum for the development, promotion and dissemination of best clinical practice for the benefit of patients through education, training, research and quality improvement. With this in mind, the Association of Coloproctology of Great Britain and Ireland (ACPGBI) was formed 30 years ago and now has over 1000 members made up of specialist surgeons, nurses, allied medical professionals and those in training.

The Association evolved from the Royal Society of Medicine (RSM) Section of Coloproctology, which itself originated from the Section of Proctology in 1913, when Frederick Swinford‐Edwards (1853–1939) became its first President [1].

For many years the Section functioned very successfully as a colorectal educational forum, largely for the general surgeon, but remained London‐centric. There was a growing need for a new organisation with nationwide representation that would realise the political, educational and training aspirations, vital for subspecialty development, that were already beginning to evolve in the UK and for which the Section was unable to provide.

In the USA, colon and rectal surgery had been recognised as a distinct specialty with accreditation and certification through the foundation of the American Board of Colon and Rectal Surgery in 1949 [2]. The early vision in the UK was to form an equivalent British Council of Coloproctology, but based within the RSM. Between 1985 and 1988, informal discussions were held between the RSM Council and the Section, represented by Philip Schofield, Bill Heald and Adam Lewis, the so‐called ‘gang of three’, who were all committed specialist colorectal surgeons. The RSM Council rejected the proposal after protracted discussions.

The concept of an independent specialist society was a pivotal development following a teaching day in November 1987 at the George Hotel, Edinburgh. Here the ‘gang of three’ expanded into a ‘gang of five’, with the addition of Charles Mann, the RSM Section President, and Roger Grace, President and Honorary Secretary of the RSM Section. There were discussions over the following year at Section Council meetings about forming a national society within the RSM and a working party was established on 20 June 1989. Geoff Oates, President‐Elect of the Section, replaced Charles Mann. James Thomson, the next Honorary Secretary, and Barry Jackson, President‐in‐Waiting, were co‐opted. The working party met on a further five occasions over the following year, until the concept was finally rejected by the RSM, when John Alexander‐Williams and Bill Heald were President and Secretary of the Section. The group decided to form a Society in any case, and on 29 August 1989 the ACPGBI was born.

The new Society's inaugural meeting was chaired by Geoff Oates on 9 March 1990 at the RSM President's Day in Birmingham, with 57 founder members attending. This was purely a business meeting and Terms of Reference were agreed. The Association would be multidisciplinary, with representation from all regions of the UK and the Republic of Ireland, provide education, advise on all medico‐political issues pertaining to coloproctology and foster international relations.

**Figure 1:**
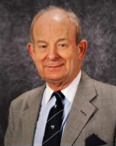
Geoff Oates, first ACPGBI President (1990–91)

There was another founding meeting in the autumn of 1990 at the General Hospital in Birmingham when Geoff Oates was elected as the ACPGBI’s first President (Table 1, Figure 1), along with Norman Addison (President‐Elect), James Thomson (Honorary Secretary) and Chris Marks (Assistant Secretary) (Table 2). Roger Grace was elected Honorary Treasurer (Table 3). The RSM Section and the ACPGBI met on consecutive days in Harrogate in June 1991.

**Table 1:** Presidents of the ACPGBI

President	Years of office
Geoff Oates	1990–91
Norman Addison	1991–92
Philip Schofield	1992–93
Peter Hawley	1993–94
Liam Kirwan	1994–95
Bill Heald	1995–96
Roger Grace	1996–97
George Parks	1997–98
Sir Miles Irving	1998–99
John Nicholls	1999–2000
Peter Lee	2000–01
Chris Marks	2001–02
Neil Mortensen	2002–03
Mike Thompson	2003–04
John Hyland	2004–05
Andrew Shorthouse	2005–06
Paul Finan	2006–07
John Northover	2007–08
Nick Carr	2008–09
Najib Haboubi	2009–10
Mike Parker	2010–11
Nigel Scott	2011–12
Graham Williams	2012–13
Karen Nugent	2013–14
Asha Senapati	2014–15
Bob Steele	2015–16
Peter Dawson	2016–17
Jim Hill	2017–18
Brendan Moran	2018–19
Nicola Fearnhead	2019–20
Steve Brown	2020–21

**Table 2:** Honorary secretaries

Honorary secretary	Years of office
James Thomson	1990–91
Chris Marks	1991–92
Adam Lewis	1992–94
Bob Lane	1994–96
Andrew Shorthouse	1996–99
Mike Thompson	1999–2002
Paul Finan	2002–05
Nigel Scott	2005–08
Karen Nugent	2008–11
Adam Scott	2011–14
Steve Brown	2014–16
Pete Sagar	2016–18
Charles Maxwell‐Armstrong	2018–20
James Wheeler	2020–22

**Table 3:** Honorary treasurers

Honorary treasurer	Years of office
Roger Grace	1990–93
Mike Thompson	1993–98
Paul Finan	1998–2002
Nick Carr	2002–07
Mike Parker	2007–10
Jonathon Reynolds	2010–13
Peter Dawson	2013–15
Balgit Singh	2015–17
Timothy Cook	2017–19
Seamus Kelly	2019–21

Financial accounts from the first year detail an income of around £8000 and an expenditure of £1500. In comparison, the most recent accounts after three decades show an income of almost £500 000 and expenditure of just under that figure.

## The constitution

The original constitution of 1990 defined Council structure. The Executive consisted of the Officers and Chairs of the main committees (see below). Chapter representatives from the regions throughout the UK and Ireland were a link between Council and the grassroots membership, and were responsible for organising local clinical and business meetings. As a multidisciplinary society, representatives from affiliated nonsurgical societies were invited onto Council. A clinical scientific and annual general meeting (AGM) to be held every year was agreed. The President served for 1 year, although the Secretary and Treasurer were elected for longer periods to allow for continuity.

From 1991 the Council met regularly on two or three occasions throughout the year and additionally at the Annual Meeting. An Education Committee was established from the outset with the Research and General Purpose Committee (subsequently becoming the Multidisciplinary Committee) established a few years later (Tables 4, 5, 6).

**Table 4:** Research and Audit Committee

Chair	Years of office
Neil Mortensen	1995–98
John Northover	1998–2001
Asha Senapati	2001–06
John Scholefield	2006–10
David Jayne	2010–13
Nicola Fearnhead	2013–17
Austin Acheson	2017–20
Jim Tiernan	2020–

**Table 5:** Education and Training Committee

Chair	Years of office
Philip Schofield	1991–96
John Nicholls	1996–99
Nigel Scott	1999–2003
Jim Hill	2003–06
Graham Williams	2006–10
John Hartley	2010–13
Justin Davies	2013–16
James Wheeler	2016–19
Laura Hancock	2019–

**Table 6:** Multidisciplinary Care Committee^a^

Chair	Years of office
Chris Marks	1995–2000
Ian MacLennan	2000–03
John Monson	2003–07
Basil Fozard	2007–10
Brendan Moran	2010–13
Charles Maxwell‐Armstrong	2013–17
Ciaran Walsh	2017–20
Michael Davies	2020–

^a^ Formerly known as the General Purpose Committee.

As the influence and complexity of the ACPGBI grew, the Executive began in 1996 to discuss the expanding agenda in detail before each Council meeting. A new committee, chaired by Philip Schofield, was formed the same year to oversee public relations and ethics (Table 7). The Executive subsequently needed to meet several more times during the year, making the job of an Executive member quite onerous and a difficult balance to achieve between local clinical, national administrative commitments and home life. The size of Council has gradually expanded over the years but reached a peak in 1997 when 10% of the membership held elected and co‐opted Council positions. Sir Miles Irving, in 1998, was the first President who tried to reverse the trend by abolishing a significant number of posts he deemed superfluous.

**Table 7:** External Affairs Committee^a^

Chair		Years of office
Philip Schofield	1996–2000	
Roger Grace	2000–01	
Michael Keighley	2001–05	
Mike Saunders	2005–12	
Mark Coleman	2012–16	
Mark Chapman	2016–19	
Shahab Siddiqui	2019–	

^a^ Formerly known as the Public Relations and Ethics Committee.

Several constitutional revisions have been undertaken to address the increasing complexity of the organisation, expanding the document from an original seven pages to double the size with a much less generous font!

## Coat of arms and presidential chain of office

**Figure 2:**
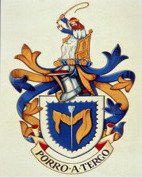
The coat of arms

**Figure 3:**
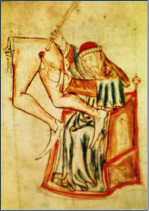
John of Arderne

During these early days there was a sense of tradition that was instilled into the Association by the previous generation and this was epitomised by three developments that endure to this day. The first was the Association coat of arms (Figure 2). Andrew Shorthouse was approached to submit a traditional design which was approved at the AGM in Harrogate in 1991. The crest featured John of Arderne, a medieval surgeon from Newark and the ‘father of coloproctology’, representing the evolution of the ACPGBI from its origins in the Section (Figure 3). In both, the patient hosting the fistula in the original Elizabethan depiction is missing, other than a ‘*sequere me*’ probe disappearing into an external opening and John of Arderne's ‘*digitus index sinister’* at the internal opening (Figure 3). The College of Arms described the left index finger as ‘imbrued’ (stained), and were not made aware of where it had actually been. The shield comprised a golden maunch, a medieval sleeve which is a traditional heraldic device and coincidentally colonic in shape. A scalloped silver border suggests colonic haustrations. James Thomson, Honorary Secretary at the time, was clearly keen on a St Mark's link and requested a Lockhart–Mummery probe to replace the (aesthetically more pleasing) Brodie's version in the shield centre. The motto, an heraldic pun, was conjured (in English) during the RSM overseas visit to Budapest and Vienna in 1991, while in transit on the Danube. Andrew Shorthouse arranged a translation by his son's Latin master. Translated, ‘*Porro a Tergo’* is, aptly, ‘*Forwards from Behind’*. Recognition by the College of Arms, London, in the form of Letters Patent granted in 1994 was expensive (it would cost £13 500 today) but unanimously agreed by Council.

**Figure 4:**
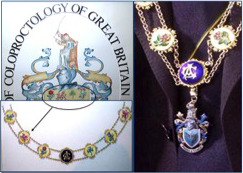
The presidential chain of office showing the national emblems taken from the coat of arms

The second ceremonial marker, worn by every President since 2002, was the gold‐enamelled presidential badge, struck in Sheffield in 1994, and a chain of office (Figure 4), commissioned later in Birmingham and costing around £4000 (about £8000 in today's prices). The badge features the coat of arms. The chain has sequential national emblems of England, Scotland, Wales and Ireland. These national emblems were symbolically important for the ACPGBI. Whilst both developments might now be considered anachronistic, the coat of arms currently remains the internationally recognisable symbol of the ACPGBI.

The third is a lapel badge incorporating the coat of arms, worn by Past Presidents at the Annual Meeting and Past‐Presidents’ Dinner.

## Annual meetings

**Figure 5:**
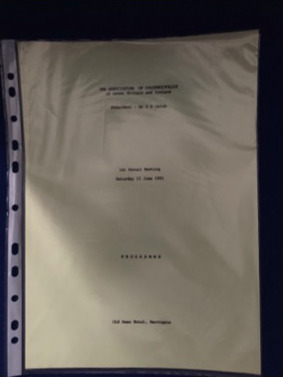
Programme for the first Annual Meeting (Harrogate 1991)

The first official Annual Meeting was held at the Old Swan Hotel in Harrogate on 15 June 1991, when Geoff Oates handed over the presidency to Norman Addison. The very first ACPGBI paper, on anorectal manometry, was given by Paul Durdey in a plenary session chaired by Roger Grace. Other presentations were by future prominent members including Nigel Scott, John Scholefield and Dion Morton. The single‐day meeting was attended by 107 of the 240 signed‐up members. The programme was a word‐processed, cyclostyle‐printed, hand‐stapled six pages of green A4 without any listed sponsors (Figure 5). In contrast, the Dublin meeting in 2019 was attended by more than 1000 delegates over 3 days, with an online programme, prominent sponsor advertising and 28 pages of content. Over the past 30 years the meeting has gradually metamorphosed into today's modern format.

The second Annual Meeting, held near Southampton, was a full day with the Friday afternoon entirely devoted to the AGM. It had a more sophisticated programme all contained in a plastic folder. We suspect there would be a slightly smaller audience for a 2.5‐h AGM marathon today.

Further meetings saw an expansion in delegate numbers and venue size. The 1994 meeting, held at the RSM in Peter Hawley's presidential year, was the first of the 3‐day meetings and the AGM remained a plenary 2‐h session. Other notable early Annual Meetings included the first to be held in Scotland (Glasgow 1993) and Ireland (Cork 1995), hosted by Philip Schofield and Liam Kirwan in their respective presidential years. A Westerly Oceanlord, chartered and skippered by John Quayle with an ACPGBI crew of five was preferable to Ryanair and the conference hotel, and no more expensive after sharing costs including an add‐on to Fastnet and the Scillies! Problem cases were liberally discussed to justify the trip.

In 1997 the ACPGBI held a joint meeting in Edinburgh with the European Council of Coloproctology (ECCP). The ACPGBI Programme Committee, chaired by Roger Grace in his presidential year, maintained tight control of the scientific content and speaker selection. The final programme had not been endorsed by the ECCP, straining relations to some extent.

An exacerbation of the conflict in Northern Ireland, prior to the Good Friday Agreement in April 1998, forced cancellation of George Parks' Annual Meeting in Belfast in his presidential year. The meeting was rearranged at very short notice and was held at the Grand Hotel, St Helier, Jersey. To date, no Annual Meeting has been held in Northern Ireland. A reflection of the time was the fact that the trade exhibition for Jersey was planned with squares and oblongs sketched on graph paper depicting stand size and location for each exhibitor as far away from competitors as possible. The Honorary Treasurer's (Paul Finan) children, in prelaptop days, cut the shapes and juggled them around on the Sunday before the meeting began. Of note the first Past‐Presidents' Dinner was held at this meeting.

Meeting the cost of a 3‐day event in a major conference centre for increasing numbers of delegates year on year, and providing a larger trade exhibition area, was becoming a significant financial challenge. By 1999, ACPGBI capital reserves had not reached the level whereby the cost of an unforeseen meeting cancellation, for example due to a natural disaster or pandemic, would be covered. Sir Miles Irving, a native of Southport, negotiated remarkably favourable rates with the local council to hire the elegant art deco Floral Hall, where over 400 delegates and a large trade contingent attended a very good scientific meeting. This significantly consolidated the Association's finances. The Southport meeting was also memorable for evening entertainment, with free roller‐coaster rides at the local amusement park along the sea front.

Ideally Annual Meetings were arranged to be in diverse locations across the UK and Ireland, for example Glasgow, Edinburgh and Dublin. In recent years local costs and less favourable exchange rates in Ireland have become a major financial challenge for planning meetings there, but the constitutionally bound Anglo‐Irish relationship remains of paramount importance to the ACPGBI. Irish meetings remain extremely popular with the membership. Several Presidents have been in receipt of exceptional Irish hospitality with invitations to Chapter meetings and dinners, the latter not often remembered but the former seldom forgotten.

As the Society expanded, venue choice with the necessary capacity for delegates and a trade exhibition became more limited. With notable exceptions (Brighton 2000, Manchester 2002, Gateshead Sage 2006, Bournemouth 2010 and 2017) the meeting has rotated through large conference centres in Birmingham, Harrogate, Liverpool, Edinburgh and Glasgow. There has been no meeting thus far in Wales. Contained hotel complexes away from major conurbations have been considered in the past as a means of diversifying the geographical location of the annual meeting. The restrictions associated with limited transport links and urban infrastructure have caused issues with both sponsors and the general ethos of the meeting.

In 2015 a new meeting format was trialled, based upon the Digestive Diseases Week model, after recurrent consideration at Executive meetings over a period of 25 years. Initially sentiment was mixed and largely negative. The concern was that it may be financially disadvantageous and that the ACPGBI would risk losing its identity. The Association finally joined forces with the British Society of Gastroenterology (BSG), the Association of Upper Gastrointestinal Surgeons (AUGIS), the British Association of Parenteral and Enteral Nutrition (BAPEN) and the British Association for Study of the Liver (BASL) to hold a Digestive Disorders Foundation (DDF) meeting in London. Although it was a financial success, there was a significant reduction in attendance by registered ACPGBI members. Many preferred the format of a stand‐alone ACPGBI meeting, which resulted in withdrawal of the ACPGBI from future joint ventures with the DDF.

A strict policy of successive Honorary Treasurers was to build and maintain sufficient reserves to cover the cost of a failed Annual Meeting. Cancellation penalties are expensive. It is difficult to insure for some ‘acts of God’ such as pandemics or terrorism. In June 2007, a green Cherokee jeep full of propane canisters rammed the entrance to Glasgow International Airport, setting it alight [3]. This was a serious threat to the Glasgow meeting days later. Two years earlier, the Dublin Tripartite coincided with the London 7/7 terrorist bombings. On that occasion all flights from Dublin to the UK were cancelled, ensuring high attendance to the very end of the meeting [4].

More recently the COVID‐19 pandemic forced cancellation of the Edinburgh Annual Meeting planned for July 2020. The financial impact is still under review but will be substantial. The President, Nicola Fearnhead, showed exemplary leadership during the crisis. Some online conference content was made available as a virtual resource and may set the template for future ‘hybrid’ meetings.

## Appointment of an administrator

**Figure 6:**
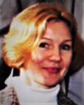
Anne O’Mara. ACPGBI Administrator and recipient of the Geoff Oates medal

Initially all Association business and the Annual Meetings were organised in‐house with the assistance of a junior administrator seconded from the Association of Surgeons. This was facilitated by a move to a new office within the Association of Surgeons (ASGBI) complex at the Royal College of Surgeons in January 1994. The benefit to the ACPGBI was the shared membership database managed by the ASGBI. The amount of secretarial assistance in the College was largely inadequate and much of the work of the Honorary Secretary and other Executive Officers was delegated to their own personal NHS secretaries to meet the shortfall. By 1996 the ACPGBI was expanding rapidly in terms of its membership and outgrowing its infrastructure and activity. As the complexity grew, the indication for more dedicated and professional administrative help became overwhelming. Andrew Shorthouse, the ACPGBI Honorary Secretary (1996–99), had previously held the same post at the British Association of Surgical Oncology (BASO), where there was a highly efficient full time administrator. Anne O’Mara, a junior administrator from the Faculty of Dentistry, was highly recommended for the position with the ACPGBI and was appointed as a full‐time administrator in late 1997 (Figure 6). A significant rise in the annual subscription rise of 40% (to afford her appointment) was nevertheless unanimously supported at the AGM. It was a sound investment. She proved to be a faithful, highly effective, efficient, reliable and approachable servant of the Association for over 20 years until her retirement in 2019. As the only continuously serving member of the Association during this time, Anne was a most reliable source of corporate memory. She was replaced by Adele Sutton who already had many years efficiently assisting Anne with the Annual Meeting.

Organisation of the Annual Meeting was Anne's responsibility for many years. The pressure on her at this time of year was immense and, as many Honorary Secretaries will testify, tension increased as July approached. In 2014 it was agreed that an events organiser should be employed to ease the pressure. Edinburgh‐based Integrity International Events Ltd was appointed after a tendering process. Integrity was already the administrator and conference organiser for the European Society of Coloproctology (ESCP) and its predecessor the European Association of Coloproctology (EACP). Despite the significant extra cost this move was considered good value. Other than the DDF meeting in 2015, Integrity has organised all the meetings since that time. In 2020 the company successfully retained the contract until 2024 when a further tender is planned.

Alice Brook was appointed in 2015 as an assistant to Anne O’Mara. This additional administrative post continued with the appointment of Nicole Taub who has particular skills in social media management, so important for rapid dissemination of communications, particularly education and research. Encouragement of younger consultants and trainees to become actively involved with ACPGBI activities through social media is vitally important for the future of the organisation.

## Officers of the association

The Officers of the Association are the President, President‐Elect, President‐in‐Waiting (1‐year term each), Honorary Secretary and Honorary Treasurer and their assistants [5], and the Chairs of the various committees (Tables 1, 2, 3, 4, 5, 6, 7). There have been several unsuccessful attempts to increase the Presidency to a 2‐year term; but most sense there is ample opportunity to work over 3 years ‘across the presidencies’ to achieve strategic goals.

**Figure 7:**
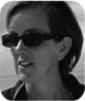
Karen Nugent, the first female President of the ACPGBI (2013–14)

**Figure 8:**
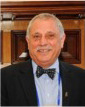
Najib Haboubi, the only nonsurgical President of the ACPGBI (2009–10)

To date the ACPGBI has elected 31 Presidents [5] (Table 1). The ACPGBI is a strong advocate of equality and diversity. It is laudable that over the last 8 years, there have been three female Presidents beginning with Karen Nugent in 2013–14 (Figure 7), then Asha Senapati in 2014–15 and Nicola Fearnhead in 2019–20. Najib Haboubi (Figure 8) was elected in 2008–09 as the only President without a surgical background. His histopathological and political contribution to ACPGBI scientific and political activity, and his role in the evolution of the ESCP over many years, was huge. Whilst originally from Yorkshire, Nick Carr has been the only President from a hospital in Wales and Bob Steele, based in Dundee, the only President to represent Scotland. Liam Kirwan was the first President from the Republic of Ireland and George Parks represented Northern Ireland. Both Liam Kirwan and Peter Lee went to Penang after their presidencies, spreading the ACPGBI word in Southeast Asia!

In October 2011 the ACPGBI investigated the WebEx teleconference system. Whilst the technology was considered potentially suitable for executive meetings it was not until the COVID‐19 pandemic in 2020, with forced social distancing, that video conferencing really became established. The Council and Executive of the time embraced the technology with a barrage of meetings, hugely facilitating access amongst Executive and Council members, at one stage several times per week. The Association has subsequently invested heavily with the intent to continue its use.

## The four main committees

### Developments in education and training

One of the underlying principles behind the formation of the Association and gaining freedom from the constraints of the RSM Section was to influence training and accreditation in coloproctology, which the Section was unable to do. It is no surprise that the education committee became such an important component of the Association from its inception. During the 1990s, and indeed up until the present day, there was a drive for recognition of coloproctology as a subspecialty with its own criteria for accreditation. ‘Design for Coloproctology’ was first released by the Clinical Services Committee under the chairmanship of Chris Marks in 1999 and has undergone two further revisions [6]. The first edition made recommendations on clinical services and staffing levels after the Calman Report [7], defining a colorectal surgeon as one dedicated to colon and rectal surgery, or alternatively with an interest in the subspecialty, but in either case playing a full part in the emergency general surgical rota.

Colorectal training units were defined as ‘specialist’ (more than two surgeons with a dedicated or major interest in coloproctology) or ‘general’ (one surgeon with a dedicated or major interest in the specialty). It was proposed that trainees should spend their final year in a specialist coloproctology unit as part of their 6‐year higher general surgical training. A detailed syllabus of subspecialist requirements was drawn up by Nigel Scott and John Nicholls, mirroring the US‐style curriculum. It was a template that was adopted readily around the UK and Ireland and also helped trainees who were studying for the European Board of Surgical Qualification (EBSQ) examination.

The Calman reforms in 1993 and the European Working Time Directive in 2004 [8] resulted in a reduction in junior doctors’ hours and a shorter training period, with the threat of not achieving adequate training within the 6‐year time frame. There was significant expansion in consultant numbers, which was also necessary to support the expansion of colorectal cancer services with the introduction of the 2‐week wait and screening. Concern about the threat of inadequate training resulted in the introduction of laparoscopic fellowships in the first decade of the 21st century. The fellowships formed a major part of the UK colorectal training scene for the best part of a decade and have produced many of the current generation of active research and training members of the Association. These developments were truly transformational for our specialty in the UK. Within 10 years UK laparoscopic surgery had gone from having one of the world’s lowest rates of laparoscopic resection to one of the highest. Credit must go to both Roger Motson for the fellowships and Mark Coleman for his work with LAPCO (see later). After more than 20 years of ongoing discussion with the ACPGBI on its submission for subspecialty recognition the Specialist Advisory Committee (SAC) finally halted the process in 2012; coloproctology remains under the general surgery SAC umbrella to this day.

The Education and Training Committee has strived consistently to improve and maintain the quality of colorectal surgical education. Courses have been approved or badged on merit. ‘Motorway’ courses, including the M62, the M54 and the M27 courses, have traditionally featured in the badging process but for many years were funded with the support of industry. In 2015 the ACPGBI began to underwrite the financial risk, particularly of the M62 course, and established a coloproctology ‘tutor’ (Nader Francis) to oversee meeting organisation, ensuring robust educational content and introducing feedback. The M62 meeting eventually morphed into the Advanced Coloproctology Course to become a stalwart of the annual Association events.

The idea of a coloproctology tutor was not new. The Association originally sponsored such a post in 2003 to assist the Royal College of Surgeons (London) with coloproctology‐related courses. ACPGBI sponsorship was withdrawn after 2 years on the grounds of excessive cost and inadequate value for its members. However, the tutor was retained by the College until 2015 with no further financial commitment from the ACPGBI.

Other more recent initiatives and innovations introduced to enhance education and training were webinars, many in collaboration with the Royal College of Surgeons of Edinburgh, badging of fellowships, significant investment in high‐quality virtual technology platforms and the latest ‘Colorectal Focus’ which aims to provide a rolling 3‐yearly programme of high‐quality continuing medical education for members and linked in with the Annual Meeting.

### Developments in research and audit

The Research and Audit Committee has been vitally important for the Association and its members so it is surprising to consider that, as late as 1997, dissolution of the committee was under consideration. Reappraisal of its role was the catalyst for action and its first major sponsorship was to launch the PROSPER randomised controlled trial led by John Northover and Asha Senapati to investigate and determine the optimal surgical management of rectal prolapse [9]. Recruitment was a problem, leading to delays of several years before trial closure, but nevertheless it remains a testament to the dedication of the trial organisers and Association members who were able to bring a truly ACPGBI‐delivered trial to fruition. The ACPGBI had injected £5000 into the trial over the initial 2 years but it was soon realised that running such a trial was possible only with the help of a generous funding body.

After the millennium there was considerable enthusiasm for more trials, leading to a proposal that the Birmingham Clinical Trials Unit should organise and conduct future trials on behalf of the ACPGBI. Not everyone agreed because other trials units would be disenfranchised. Nevertheless, the Birmingham Clinical Trials Unit continues to be linked very closely with many ACPGBI‐linked colorectal research projects.

In 2013 a modified Delphi exercise to identify the most important coloproctology research questions from the patient's perspective was proposed by Nicola Fearnhead and the Bowel Disease Research Foundation (BDRF), and enthusiastically accepted [10]. A plenary session at the DDF meeting in 2015, which involved patients, was hugely successful. Subsequent ‘Delphi games’ were organised, leading to a structured approach for future coloproctology research. A series of highly successful government‐funded trials such as CIPHER (parastomal hernia), ALLEGRO (intravenous lignocaine in postoperative recovery) and PREPARE ABC (prehabilitation for surgery) have followed.

The ‘Create’ series of regional events has acted as a catalyst for successful research projects, boosted by the creation of Surgical Site Leads to support research administration channelled through the Royal College, with Simon Bach being the first holder of this position.

ACPGBI‐sponsored audits have included malignant large bowel obstruction, transanal endoscopic microsurgery, procedure for prolapse and haemorrhoids, diverticular disease, laparoscopic surgery and the National Bowel Cancer Audit (NBOCA). In 2005 a pouch registry was developed, with two subsequent publications of accrued data [11]. The pouch registry remains the largest database of its type in the world, but in view of issues of incomplete data the registry was closed in 2019. It was replaced by a new inflammatory bowel disease (IBD) surgery database incorporating sophisticated online access for inclusion of patient‐reported outcomes. Provided that members are compliant with data entry it will become a very powerful tool for IBD‐specific audit. Another database, SWORD, works in tandem with the IBD surgery database, to provide data on pouch numbers carried out by English surgeons.

### The National Bowel Cancer Audit

The NBOCA merits special attention. Whilst the Association has been involved with this audit since June 1997, supported by Jeff Stamatakis, forerunners existed from the Northern Region, Trent/Wales, Wessex and Lothian. These audits were poorly funded but the enthusiasm and desire for accurate data collection served as a catalyst for a national audit. Initially the NBOCA was voluntary but recruitment issues led to a case ascertainment of only 30% of eligible patients. Poor risk adjustment led to discrepancies between apparent and actual quality of some high‐performance units. By 2013 it was clear that the Government was intent on publishing surgeon‐specific data. The ACPGBI was adamant that it retained control of NBOCA data on its release to ensure they were both accurate and representative. At this time national audits had been co‐ordinated by the Healthcare Quality Improvement Partnership (HQIP) and they agreed to support the national colorectal cancer audit on two conditions: an increase in case ascertainment and an agreement by the ACPGBI to work towards publication of identifiable outcomes. Surgeon‐specific outcome data were published soon afterwards to include 90‐day mortality as the main outcome, thereby including late perioperative deaths. Detailed oversight of this process by Graham Williams and his wife Angie ensured a robust process, and surgeon‐specific outcomes are now regularly published, on a rolling basis, to ensure adequate numbers and publication of credible measures. Risk stratification models were developed together with the Clinical Effectiveness Unit at the Royal College of Surgeons of England, ensuring fair presentation of data. High‐volume surgeons, whose case mortality was within confidence thresholds, could be compared favourably with low‐volume surgeons who had zero mortality. A robust policy of support for outlying units and surgeons was introduced, with the Consultant Outcome Publication now an annual feature on the website and very much at the forefront of the national audits. Credit must go to Jim Hill for leading on many of these more recent developments.

The precise definition of a colorectal cancer surgeon remains controversial. Resection with curative intent in excess of 20 colorectal cancers was first proposed in March 2007. Despite no evidence to support it, this definition persists but is currently under consideration.

In 2014 the NBOCA reported enormous variation in core outcomes. Wales was a prominent outlier compared with the individual regions in England but the data were potentially flawed. Although data collected in Wales would have allowed for risk adjustment, the Clinical Effectiveness Unit was unable to gain access to it. Chapter representatives were keen to explore other potential reasons for outlying status and the Association offered help. In 2015 an Extraordinary Meeting was convened by the ACPGBI to bring together Chapter representatives and representatives from all the Welsh Trusts to present their data and explore the discrepancies. The important outcome was a recommendation to the Welsh Assembly to properly fund multidisciplinary team (MDT) working and to emphasise the importance of strict audit data collection.

### External Affairs

The Public Relations and Ethics Committee, organised and chaired by Philip Schofield, was formed in 1996 to deal with matters concerning relations with external organisations, patient information, ethical matters and medico‐legal issues. The focus of the committee at that time was the relationship with the media and promotion of bowel‐related issues. A prominent and enthusiastic early supporter was the late Lynn Faulds Wood, who for many years had been a well‐recognised television presenter and ‘consumer champion’ (BBC *Watchdog*) and who had herself recovered from bowel cancer after surgery in 1991. She spoke publicly about her experience from the patient perspective. Lynn worked closely with the Association, its Public Relations and Ethics Committee, and particularly with Mike Thompson, throughout the Awareness of Colorectal Cancer campaign in the late 1990s. Her media experience and charisma made her a firm favourite at several Annual meetings from the late 1990s, but she was not afraid to challenge the ‘status quo’! Her view was that ‘coloproctology’ was an incomprehensible component of the ACPGBI title for the layperson and that it should be renamed the ‘Association of Bowel Specialists’. Rebranding attempts were unsuccessful and remained so on several later occasions.

An early venture was the production of patient information leaflets, a recurring theme over the next 10 years under three successive committee chairs.

Eventually the committee diversified and some years later appeared to lack purpose, but in 2011 it was revitalised. There was a need to develop and manage the website to include a system to manage subscriptions (see Relationship with the ASGBI).

### The website

In 1999, a new website came under the auspices of the Research and Audit Committee who delegated responsibility for content and maintenance to a group of enthusiastic individuals. Its importance and the need for investment were not universally agreed. In the February 2000 Council Minutes, the value of investing £5000–10 000 on website development was questioned, as even ‘members with access to the internet would not use it’! Nevertheless, the tender for a professional developer was approved, and in 2001 a Website Committee was formed ‘to serve an ongoing need for revamping’. A patient‐orientated section was introduced by Lynn Faulds Wood in 2003.

Responsibility for the website passed to various enthusiasts and designers. In 2008 an Information Management and Technology Committee was formed, led by Jason Smith. Professional help was commissioned from Mixd in 2009. At a ‘strategy meeting’ in 2010 the need for further major revision became a priority. Mark Coleman was appointed in 2012 with a brief to accelerate this agenda, in his capacity as Chair of External Affairs. Mixd was again approached to design an online registry of members, together with their interests, and remained the main contractor for a further 8 years. The ACPGBI is currently (2020) redeveloping the website with the help of LightMedia.

### Developments in multidisciplinary care

The MCC Committee covers all aspects of clinical care falling outside the remit of the other major committees. It was originally the General Purpose Committee and then renamed the Clinical Services Committee; in 2010 it became the MCC Committee to reflect the multidisciplinary makeup of the various subcommittees created in 1999. It is now an extremely important committee which has expanded over the years to provide direction for a multitude of subcommittees involved with IBD, cancer, colonoscopy and pelvic floor surgery. More recent subcommittee additions include peritoneal carcinomatosis, robotics, proctology and abdominal wall. Although at first sight such a large number of subcommittees might appear unwieldy, it proved to be of great benefit to the membership because of the breadth of expertise it could offer. Co‐ordination of so many members contributing to each subcommittee is a major challenge for the MCC chair. Furthermore, the Clinical Governance Board, which offers support to members in trouble, falls under the MCC remit.

One of the first tasks of the MCC was to co‐ordinate guidelines, sometimes in unison with the National Institute for Health and Care Excellence. Over the years various topics have been covered, including endoscopy, management of colorectal cancer (COG), diverticular disease, IBD, fissure and fistula [12]. In addition, it drew up the requirements for a coloproctology service in ‘Design for Coloproctology’ [6].

Interestingly, minutes from some Council meetings in the early 1990s include discussion on laparoscopic resection and clearly illustrate the initial reticence of Council in its endorsement, in respect of colorectal cancer resection, without the support of randomised controlled trial (RCT) data. This was a major factor which contributed to the delay in adopting the technique nationally, compared with other countries in Europe and the USA. The earliest description of a laparoscopic approach to colorectal cancer resection was in 1991 [13]. Even by 1997 it was restricted in the UK to study populations in specialist centres, in stark contrast to the speed with which laparoscopic cholecystectomy became established, initially without RCT evidence for efficacy. Its clear superiority and safety did not require additional confirmatory RCT evidence. The first RCT of laparoscopically assisted resection for colorectal cancer was published in 1997 [14], but failed to provide the necessary robust evidence for its safety and effectiveness when compared with open surgery, the holy grail craved by Council before they would support its use nationally.

Also in 1995, the ACPGBI Council would not support a course on laparoscopic rectopexy, proposed by Lord Darzi, ‘on the basis of the unproven science’. Review of subsequent minutes reflects a slow acceptance by the ACPGBI Council over a number of years, but support gathered momentum during Mike Thompson's presidency in 2003, when laparoscopic fellowships were introduced as part of the accreditation process of training units and trainees. Support for the LAPCO initiative was seminal in allowing retraining of older established consultants [15]. Final acceptance of laparoscopy into mainstream practice occurred in 2010 when MDTs were instructed to ensure that every patient suitable for laparoscopic resection was offered this choice of treatment.

A further area of historical interest was a proposal that surgeons trained in total mesorectal excision (TME) should be identified and in some way accredited. This was countered initially by some influential members of Council ‘as the operation had yet to be defined … with insufficient scientific (RCT) trials’. ‘Indignation’ was expressed by others.

Around the millennium the Government introduced an initiative to direct resources specifically to cancer services and MDT management. With an emphasis on improving access and public awareness of bowel symptoms, the pressure on outpatient and inpatient services inevitably increased. The ACPGBI published ‘Resources for Coloproctology’ detailing a wide range of unit recommendations [16]. Consultants of all disciplines readjusted job plans to facilitate the extra workload and MDT attendance. For surgeons, recommendations were 1.7 operating sessions, two outpatient clinics and 0.75 endoscopy sessions per week. Five full‐time equivalent colorectal surgeons were recommended to serve a population of 500 000 and perform a challenging minimum of 250 colonoscopies per year. Although some of the methodology in deriving these figures was questionable, the staffing consequences of the 2‐week wait, increased pressure on colonoscopy services, the need for more specialist nurses and essential nonsurgical services such as radiology, pathology, oncology and palliative care were correctly anticipated. Introduction of the new consultant contract prompted a further update of ‘Resources for Coloproctology’ in 2006 and again in 2015, when methodology was far more rigorous. There was significant expansion in detail to provide a robust reference for current resource requirements for all colorectal units [17].

There have been several examples of important interactions between the main committees. For example, extralevator abdominoperineal excision (ELAPE), initiated in 2010, was supported by the MCC and Education and Training committees working together. A more recent example in 2017 was transanal total mesorectal excision (TaTME), tabled as an area in which the ACPGBI might take a lead. The need for a robust audit platform, together with a national training programme for its structured introduction into practice, required the collaboration of Education and Training, Research and Audit and MCC committees. In contrast with ELAPE, the Executive as a whole remained cautious about their endorsement of such a procedure in its early stage of development. That anxiety was justified by 2020 when its safety was questioned. An updated consensus document was published on behalf of the Executive, emphasising the need for appropriate governance and continued structured training if the procedure was to continue playing a role [18]. This demonstrates the difficult position in which the ACPGBI is sometimes placed: on the one hand not wishing to support potentially unsafe or ineffective innovation but on the other wishing not to delay progress in patient care.

IMPACT is another very recent example of intercommittee collaboration, with a programme of events aimed at improvement in education and care for those managing advanced colorectal cancer.

It is important to acknowledge the support received from Professor Sir Mike Richards, the first National Cancer Director, for the MMC‐led cancer‐orientated projects such as TME training and LAPCO. He really helped to push the agenda forwards.

## Other council representation

### Patient Liaison Group

During the early years of the Association there was minimal patient involvement in the production of policy documents. Lynn Faulds Wood was consulted on numerous occasions during the late 1990s and her role in promoting bowel cancer awareness, co‐ordinated through the Public Relations Committee, was invaluable. However, as part of strategy reorganisation after the conviction of Harold Shipman, the Royal College of Surgeons of England, keen to support the various specialty associations, established a Patient Liaison Group. Jo Church was introduced to the ACPGBI Executive for the first time in February 2008. Over the next 7 years, until her retirement in 2015, she was invaluable in establishing a formal Patient Liaison Group within the Association, which she chaired. The group expanded to become one of the jewels of the Association, maintaining a strong patient focus on every issue that arose.

### Chapters

Regional Chapters were active from the very origins of the Association. The first meetings were held in 1991. The general geographical structure of these Chapters persists to this day but there have been challenges for individual Chapter representatives and successive Honorary Secretaries. Vagaries include transfer of hospitals near the boundary to an adjacent region and the distances needed for Chapter members to travel across some of the larger regions in the UK and Ireland. Nevertheless, over many years regional meetings have been invaluable for the education and networking of members at a more local level, enabling them to feel valued as members of a vibrant organisation, for the opportunity to present data and share clinical problems in a semi‐formal setting and exchange ideas. Chapter representatives are an important channel of communication between Council and its grassroots membership, and form the backbone of Council meetings to feedback proposals from the Executive and resist any reforms that the membership consider to be too radical. The view of the membership is of paramount importance and voiced by voting at the AGM.

### Trustees

The Executive and Council have always been accountable to a group of Trustees who were initially members of the Executive. This was formalised when the ACPGBI incorporated as a company limited by guarantee with charitable status in 2007–08. In 2016 a separate Board of Trustees was formed which was given considerable impetus with the appointment of an able Chairman in Bob Steele and some influential lay members who oversee governance and give assistance and advice to the Executive Committee, and particularly the Honorary Treasurer, in properly executing the charitable aims of the organisation. Whilst adding to an already complex bureaucracy, the formality of its structure, in any case a legal requirement of incorporation, ensures transparency of the Association in pursuance of its charitable objectives.

### The Bowel Disease Research Foundation (BDRF)

The importance of the role of the ACPGBI in supporting research was evident when the BDRF became its research charity arm. The foundation was unique in bringing together researchers, scientists and practitioners in a patient‐orientated collaboration, but its formation was a struggle.

Whilst first proposed and registered as a charity in the early 1990s and pump‐primed with a donation of £1000 from the ACPGBI, the research foundation struggled to raise funds and needed members’ covenant donations for several years. Issues with funding continued through the millennium. It was clear that much larger donations were needed to support research grants and the appointment of a dedicated professional fundraiser was essential. Agreement was eventually reached with the ACPGBI to support the BDRF with surplus profit from the Annual Meeting from 2003 onwards. By 2005 reserves had grown to an extent whereby research grants became available to ACPGBI members.

The resultant drain on ACPGBI meeting profits catalysed the search for a dedicated fundraiser, which was in the interests of both parties. In 2006 the ACPGBI supported the appointment with a grant of £84 000 over 2 years. The relationship between the two registered charities was complex but the arrangement was seen to be mutually beneficial. Although there was an urgent need for research foundation investment, it also presented a significant financial risk to both in the event of failure. In December 2006, Martyn Hall was appointed and a business case submitted to the ACPGBI. The name ‘Bowel Disease Research Foundation’ was agreed, but only after much argument. Even then the minutes refer to its naming as ‘unattractive’. In comparison with the speculative but pivotal decision to employ a full‐time ACPGBI administrator 10 years earlier, Martyn’s appointment was also a huge success. He consistently met fundraising targets set by the Association year on year and strengthened the BDRF as a core bowel disease research foundation charity. Grant applications followed, and within 3 years over 30 applications were occurring annually. The Research and Audit Committee acted as the advisory committee for grant adjudication, and its recommendations were strictly adhered to by the BDRF Trustees, thus ensuring scientific validity and subsequent endorsement by the National Institute for Health Research. Peter Rowbottom took over as CEO in May 2016. The ACPGBI continued to support the BDRF but reduced its funding year on year as it became more secure and financially independent, boosted by a substantial legacy in 2011. The charity continues to support ACPGBI members with regular grants and latterly travelling fellowships specifically aimed at research quality improvement.

The BDRF has funded 100 projects across 280 locations with 400 researchers. These have produced over 80 publications in peer‐reviewed journals and many projects have gone on to receive further funding for larger clinical trials. In total, over £3 million worth of research has been funded by the BDRF.

In 2018 a merger of the BDRF with the charity Bowel and Cancer Research was proposed and unanimously supported by the Executive as a positive development. The merger has now occurred, and at the time of writing the new charity name is an extremely well‐kept secret. Links with the ACPGBI are intended to be not only continued but enhanced, and a successful merger will undoubtedly secure the long‐term future of the BDRF.

### The Nurses’ Forum

The proposal for nurses to become affiliate members was tabled as early as 1992 but did not gain momentum until 3 years later. Many felt that only stoma therapists should be invited and not nurse specialists. Council initially rejected the addition of a nurse representative but the climate changed dramatically, fuelled by the enthusiasm of stoma nurses in particular. A Nurses’ Forum was established and affiliate membership swelled. The inaugural meeting of the Nurses’ Forum, chaired by Terrie Porrett, took place in April 2000.

Unfortunately the ACPGBI currently struggles to engage all nurses with an interest in coloproctology. The Forum was weakened by the subsequent establishment of other nurse‐led societies, especially the Association of Stoma Care Nurses, the World Council of Enterostomal Therapists and the BSG‐affiliated Nurses’ Association. Proposals for links with the other societies and even reciprocal membership have so far failed to increase ACPGBI membership. When many specialist nursing posts were sponsored by industry, their attendance at the Annual Meeting was high. However, as Trusts took over contracts, nurses were pulled in different directions (e.g. 2‐week wait clinics and ‘straight‐to‐test’ services) with the result that numbers fell. Nevertheless, the Association acknowledges the important role of colorectal nurse specialists in MDT management and remains committed to their support with planned initiatives likely to increase membership.

### The Pelvic Floor Society

The concept of a pelvic floor subgroup was originally suggested in 1996 but not taken forward. The idea was again tabled in 2006 and a functional bowel disorder subcommittee was established the following year within the Clinical Services Committee. Endoanal ultrasound (EAUS) training followed and has remained a regular feature of the Annual Meeting to this day. The Pelvic Floor Society was formally established in 2013, due to the popularity and success of earlier informal meetings where attendance by interested surgeons, physiologists and radiologists was huge. Based on this success, a separate specialist society independent of the ACPGBI had been proposed but failed to achieve the necessary support. The Society therefore remained within the ACPGBI but by this stage was considered too large to be a MCC subcommittee. Constitutional changes permitted a separate Executive within the ACPGBI umbrella. A website was established in 2015 and the Society rode the crest of a wave before the pelvic mesh controversy destabilised the group in 2017. But able management by Andy Williams, who chaired the group at that time, averted catastrophe. Risk management, with carefully worded consensus statements, enhanced vigilance and accreditation to set a template against future problems.

### The Dukes’ Club

In 1997 the Executive wished to highlight the importance of trainees to the Society. At the joint ACPGBI and ECCP meeting in Edinburgh that year the Dukes’ Club was formed and its first President was John Stebbings. It was a popular initiative, particularly with the addition of a sponsored annual 2‐day educational event.

The Dukes’ Club went from strength to strength, incorporating its own website, training opportunities and sponsored fellowships. The success was so immense that the Club began to drift away from the auspices of the ACPGBI. In the last 5–10 years close dialogue has resumed, helped by the invite of a Dukes’ representative onto Council and the involvement of trainees in most aspects of Council function; many committees and subcommittees now have a Dukes’ Club representative. Trainees are the next generation of colorectal surgeons and vitally important for the future of the ACPGBI. The shift by the Executive and Council towards more integration into Association affairs, although somewhat belated, is most welcome.

The focus of the Dukes’ Club has been trainees. Newly appointed consultants, however, need support, especially with shortened training and reduced hours compared with the situation 30 years ago. The first few years in practice are stressful. Opportunities for elected positions on the various committees and subcommittee at this stage of professional development are few, for many reasons: experience is too short to have built a reputation, job pressures inhibit national involvement, and work/life balance with young family commitments. The Executive and Council, conscious of the need to represent all members, established the Early Years Consultant Network in 2020 to provide support and opportunities for early involvement in ACPGBI activities. There is a huge untapped potential for young consultants and trainees to involve themselves in audit and research, as has occurred to great effect in the ESCP.

## The association journal (*colorectal disease*)

An Association is stronger for having its own journal to encourage high‐quality research and being a voice for its members. Initially the ACPGBI was affiliated to the *International Journal of Coloproctology*, with a reduced subscription cost on proof of membership.

The desire for the Association to own its journal was a protracted process in the 1990s. There were issues with publishers and sensitivities surrounding the extent of a European presence and choice of Editor. A contract was finally agreed with Blackwell Publishers UK, with a 50% profit share between the Association and the publisher, to launch *Colorectal Disease* in 1999.

**Figure 9:**
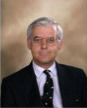
John Nicholls, President 1999–2000 and first Editor‐in‐Chief of *Colorectal Disease*

The first edition echoed the ambition of its first Editor‐in‐Chief John Nicholls (Figure 9), who established a truly international presence and readership; all eight original articles were by authors from outside the UK. Important milestones during John Nicholls’ tenure included achieving a citation profile in 2002, an impact factor of 2.059 in 2008, reaching profitability and a move to monthly publication in 2009. The 2019 impact factor of 2.997 illustrates the astonishing success of the journal over a relatively short period of time.

Neil Mortensen took over as Editor‐in‐Chief in 2015, consolidating its European credentials and improving the citation index. An inevitable decision was to publish online only in 2019, when Neil Smart became Editor‐in‐Chief. His mission is to steer the journal ‘for patients, to publish research that benefits them; for readers, to publish research that helps inform their everyday practice by focusing on benefit for patients; and for authors, to deliver a high quality and fair peer review process in a timely fashion’ [19].

John Nicholls’ ambition was to create an international multidisciplinary journal that would be attractive to other European societies, who might affiliate with it. In 1999 the forerunner of the ESCP, the EACP, adopted *Colorectal Disease* as its own official journal at preferential rates for its members, but without any other financial commitment. Joint ownership was discussed in 2003, with the ESCP after its formation in 2006 and again in 2010. There was unanimous support from the ACPGBI Executive over that period. By December 2016, supported by the Editor‐in‐Chief and the Executive, the ACPGBI President Peter Dawson offered joint ownership to the ESCP at no cost but also to share the risk, as profitability of the journal was declining. The offer was not concluded until joint ownership was revisited in 2018 by the President, Brendan Moran. By this stage *Colorectal Disease* was again profitable and the offer of co‐ownership was readjusted accordingly. The prospect of open access to the journal introduced further uncertainty about the extent of financial risk. The ESCP deferred its decision and negotiations ceased. The ACPGBI remains committed to ESCP involvement on the Editorial Board, with organisation and running of the journal, and providing future content.

## Mettings, relationships and awards

### Strategy meeting

In October 2010 an extraordinary Executive meeting was held in Oxford. The Association had been in existence for 21 years and the Executive wished to review its organisational structure to secure its future. There were over 44 serving members at the time. Mike Parker led the Council reforms with the aim of reducing its size and moving selected positions into other committees, leaving Executive representation by the four main Chairs of Office. Chapter representatives continued to serve on Council as before in order to retain the vital link with the grassroots membership. Council offered £1000 per Chapter each year to support local meetings. The Public Relations and Ethics Committee was rebranded External Affairs. Despite a recommendation that similar planning meetings should be held annually, the next was not until 5 years later when it was reconvened by the president, Asha Senapati, as the ‘Strategy’ meeting. It is now an important event for the Association as a brainstorming exercise to prioritise its future direction.

### Tripartite meeting

The Tripartite meeting grew out of a pre‐existing bipartite relationship with the Royal Society of Medicine Section and the American Society of Colon and Rectal Surgeons (ASCRS). For many years the Tripartite societies were the ASCRS, the Royal Australian College of Surgeons’ Section of Colon and Rectal Surgery with the Colorectal Surgical Society of Australia and New Zealand (CSSANZ), and the ACPGBI jointly with the Section. The ethos was international collaboration.

The first Tripartite meeting was held in Australia in 1993, then in London when Bill Heald was President in 1996. Later meetings in the UK and Ireland were held in Dublin 2005 (President John Hyland) and Birmingham 2014 (President Karen Nugent). Whilst the UK and Ireland were represented jointly by the ACPGBI and the Section, a more recent decision to invite the ESCP to participate as an equal partner in 2014 was welcomed. Deferral of the 2020 Tripartite meeting in Auckland to February 2022, due to COVID‐19, will in turn cause the planned 2023 meeting hosted by the ESCP in mainland Europe to be deferred. Other colorectal societies are emerging around the world, particularly in Asia, and the format of the Tripartite may therefore need to adjust towards even more of a global relationship. It may be that the name will need to change.

### Relationship with the Section of Coloproctology, Royal Society of Medicine

The RSM was the forerunner of the Association and in existence for more than a century. As the Association became established and evolved, the two organisations grew apart. It was originally thought that the Association would play a prominent role in the political arena and training whilst the Section focused mainly on teaching and academia. However, the expanding role of the Association in all these areas has resulted in a contraction of the Section's influence. A further problem lies in the fact that the Section does not represent the entire nation and cannot speak for coloproctology as a whole. It also tends to be London‐centric. A proposal for it to represent the London Chapters was rejected.

Financial support for Section trainee travelling fellowships has significantly depended on a risk‐free profit share from meetings held jointly with the ACPGBI, including the Tripartite. The ACPGBI Annual Meeting in Edinburgh in 1997, held jointly with the Section and the ECCP illustrates this well. The Section, as joint host, was unable to identify funding for the conference centre and other meeting charges but nevertheless received 50% of the meeting profit. The ACPGBI Council considered this an ‘unreasonable’ position but that it would be ‘churlish to undermine relationships’.

There are ongoing joint ventures such as the Goligher Lecture, Goligher Medal and the Tripartite meeting, but realistically the Section's long‐term future as a stakeholder would seem uncertain.

### Relationship with Ireland

The very first Council Meeting in 1991 minuted a preference by the Irish coloproctologists to form a Chapter of the Association rather than pursuing a separate European affiliation. However, the expanding global influence of the ESCP and Ireland's position in the EU has led to a decline in ACPGBI membership. Nevertheless, the Executive remains committed to the relationship with its very close ties.

### Relationship with the ESCP

It is clear from Executive, Council and AGM minutes and other publications that the years 1999–2006 encompassing the formation of the ESCP, and for 3 years afterwards, were somewhat turbulent [20]. There were challenging issues surrounding finance, decisions on where its administration should be based and a perception of excessive ACPGBI influence. The committee structure, function and constitution of the ESCP were largely drawn from the ACPGBI, but its influence would naturally decline over the years as the regional rotation of key officers became established. Pragmatism won the day over symbolism; administration and conference organisation by Integrity International Events Ltd remained in Edinburgh. The ESCP and its predecessor the EACP had already formed close relations with the Division of Coloproctology, Section of Surgery, Union Européenne des Médecins Spécialistes to establish, evolve and facilitate the EBSQ (coloproctology) diploma, in stark contrast to the General Surgery SAC which was, and remains, against subspecialty recognition with its own qualification and accreditation.

The importance of the ACPGBI’s involvement in the establishment of the EACP, then the ESCP, should not be forgotten; from start‐up loans in the late 1990s, a constitution template, named participation in the ACPGBI Annual Meetings and *Colorectal Disease*.

As the ESCP goes from strength to strength, a strong and close relationship with the ACPGBI is integral to the latter's own strategy for the future, not least because there are currently well over 100 members of the ACPGBI who are also ESCP members and there are many UK colorectal surgeons who are only members of the ESCP. Members from the UK have always been one of the largest constituent parts of the ESCP.

### Relationship with the ASGBI

The formation of the ACPGBI coincided with wide changes in the ASGBI itself. The emergence of subspecialties in general surgery, under the umbrella of the ASGBI, sometimes led to an uncomfortable relationship. The ASGBI, in some ways reinventing itself, needed to embrace subspecialisation and encourage collaboration and discussion around problems common to all the surgical groups. The ACPGBI, together with the other subspecialties, collaborated to everyone's mutual benefit, for instance sharing a membership database. The ACPGBI retained its office within the ASGBI complex in the RCS building in London and continued to share the database for the purposes of collecting annual subscriptions.

A recurring theme from minutes at the time of the millennium was a desire for coloproctology to be recognised as an independent subspecialty with its own SAC and direct representation on the RCS Council. This resulted in a problematic relationship with ASGBI. Furthermore, a series of documents in 2001 concerning coloproctology were supported or produced by the ASGBI with perceived minimal ACPGBI consultation.

Another area of tension was the administrative arrangement. Issues surrounding shared accommodation and subscription collection, in place since 1995, came to a head in 2012. Relations soured when ACPGBI carried out financial restructuring and review of expenditure, which led to a move away from the shared ASGBI subscription management database; slightly strained relations followed during the next few years.

There were intermittent minuted requests from the ASGBI to hold joint scientific meetings but they were rejected by the ACPGBI on the grounds of potential dilution of ACPGBI identity and loss of revenue.

Nevertheless, the current working relationship with the ASGBI is extremely convivial. It has been made stronger by the recent COVID‐19 crisis and the need to pull together to co‐ordinate advice for general surgery. The commonality of almost all members of the ACPGBI to provide emergency services has always made a harmonious relationship essential. ASGBI also provides representation on the English Royal College Council, a significant factor in maintaining this good working relationship. An example of ACPGBI collaboration, following the ASGBI’s 75th anniversary in 1995, is a session devoted to coloproctology at every ASGBI Annual Meeting. This included the virtual emergency session in 2020 to celebrate the centenary of ASGBI. From 2015, every subspecialty association under the ASGBI umbrella has sent a representative to each other's council meetings.

### Society awards

Association awards take many forms. Honorary membership has been awarded to those deemed to have made an important contribution to coloproctology, with 43 current holders.

The BJS Lectureship was inaugurated in 1996 when the first invited speaker was Glen Steele. Invitations are usually for international speakers, but there have been occasional UK exceptions.

The first John Goligher Lecture, in 1999, was given by Marvin Corman. Rotation of the lecture around the three organisations in which John Goligher had been both supportive and influential (ACPGBI, RSM Section and Tripartite) was organised by Paul Finan, with Nancy Goligher's agreement. It followed initial discussion between the ACPGBI and the Section, then with ASCRS and the Australasian organizations.

Emmanuel Tiret delivered the first *Colorectal Disease* Lecture, sponsored by the journal, in 2012. The first BDRF Lecture was given by John Northover in 2015, and more recently the inaugural Dukes’ Lecture, organised by the Dukes’ Club, was given by Scott Steele in 2019.

Travelling fellowships have been awarded to ‘early year’ consultants since 1996, the US travelling fellow being the longest established. Other fellowships have included awards to Australasian, South African and European colorectal surgeons and trainees, allowing them to visit specialist centres in the UK and Ireland.

The BJS Prize was introduced in 1996 and awarded to a trainee presenting the best paper at an ACPGBI Annual Meeting. James McCourtney was the first recipient.

In recognition of excellence, the first winner of Trainee Coloproctologist of the Year, awarded by panel and interview, was Andrew Beggs in 2016. The standard of applicants has been astounding. The irrefutable talent of many of the new generation of colorectal surgeons is very reassuring for the future.

Perhaps the most prestigious of all is the Geoff Oates Medal, in memory of the first President of the ACPGBI, and awarded for outstanding services to the Association. There have been just six recipients: John Nicholls, internationally renowned President of the Association and the driving force behind *Colorectal Disease*; George Foster for his work over two decades with the BDRF; Jo Church for her excellence as Patient Representative of the Association for many years; Graham Williams for his work with the NBOCA, his continued staunch support and his time as President of the Association; Anne O’Mara who served so admirably as administrator for nearly 20 years; and Asha Senapati who has done so much with the ACPGBI and particularly the BDRF.

### Archivist

One final development. Andrew Shorthouse was unofficially appointed (or instructed!) by Roger Grace, President in 1997, to assume the role of ACPGBI archivist. He was Honorary Secretary at the time (as if there wasn't enough to do in the pre‐Anne O’Mara days). It is he who took enormous pleasure in passing copious box files of ACPGBI memorabilia to Steve Brown, who was eternally grateful for the privilege of sorting it out!

## Conflict of interest

None.

## Author contributions

SRB: conceptualisation, investigation, resources, data curation, writing draft, review and editing, visualisation, project administration. AJS: conceptualisation, investigation, writing draft, review and editing, visualisation. PJF: conceptualisation, investigation, writing draft, review and editing, visualisation. ACPGBI Executive: review and editing.

## Appendix 1

Graham Branaghan, Michael Davies, Laura Hancock, Arthur Harikrishnan, Seamus Kelly, Charles Maxwell‐Armstrong, Peter Sagar, Shahab Siddiqui, James Tiernan, James Wheeler.
